# A New Model of RGB-D Camera Calibration Based on 3D Control Field

**DOI:** 10.3390/s19235082

**Published:** 2019-11-21

**Authors:** Chenyang Zhang, Teng Huang, Qiang Zhao

**Affiliations:** 1 School of Earth Sciences and Engineering, Hohai University, Nanjing 211100, China; zcynj@hhu.edu.cn (C.Z.);; 2 Huawei Technologies Co. Ltd., Beijing 100085, China

**Keywords:** RGB-D camera, resection space, collinear equation, relative pose, depth correction

## Abstract

With extensive application of RGB-D cameras in robotics, computer vision, and many other fields, accurate calibration becomes more and more critical to the sensors. However, most existing models for calibrating depth and the relative pose between a depth camera and an RGB camera are not universally applicable to many different kinds of RGB-D cameras. In this paper, by using the collinear equation and space resection of photogrammetry, we present a new model to correct the depth and calibrate the relative pose between depth and RGB cameras based on a 3D control field. We establish a rigorous relationship model between the two cameras; then, we optimize the relative parameters of two cameras by least-squares iteration. For depth correction, based on the extrinsic parameters related to object space, the reference depths are calculated by using a collinear equation. Then, we calibrate the depth measurements with consideration of the distortion of pixels in depth images. We apply Kinect-2 to verify the calibration parameters by registering depth and color images. We test the effect of depth correction based on 3D reconstruction. Compared to the registration results from a state-of-the-art calibration model, the registration results obtained with our calibration parameters improve dramatically. Likewise, the performances of 3D reconstruction demonstrate obvious improvements after depth correction.

## 1. Introduction

The RGB-D camera is a new type of optical sensor that was first released by Microsoft in 2009. The RGB-D camera has solved one of the main problems in the field of computer vision—a lack of depth information—by capturing depth and texture information simultaneously. Currently, because of its lower cost, smaller size, and ability to perceive depth in 3D scenes, the RGB-D camera by Microsoft Kinect has been widely applied in numerous fields such as robots [1,2,3], computer vision [4], biomedical engineering [5,6], SLAM [7], etc. An RGB-D camera is usually composed of a color camera and a depth sensor [8]. The depth sensor adopts an active imaging mode and consists of an infrared emitter and an infrared camera. In this paper, we took the Microsoft Kinect sensor, which is one of the most popular sensors on the market, as an example to introduce the principle of measuring depth and to verify our proposed calibration model.

**Figure 1:**
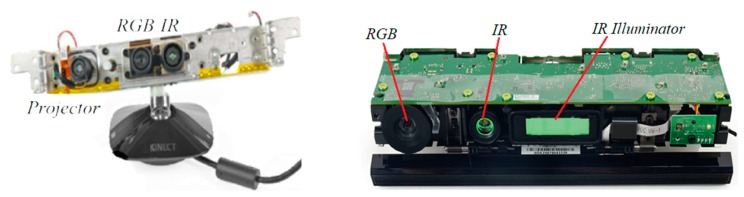
Intrinsic structure of Kinect:Kinect-1 (**left**) and Kinect-2 (**right**).

The basic structure of the Microsoft Kinect sensor contains an infrared projector, an infrared camera, and an RGB camera (shown in Figure 1). In general, the mechanism of measuring depth is based on an active imaging model, which includes the principle of structure light (SL) and time of flight (ToF) [9]. The principle of structure light is based on the theory of optical triangulation [10]. As an infrared emitter can project structure light with a certain pattern and the pattern can vary with different distances, depth can be obtained by comparing and calculating with the previous calibrated pattern. Currently, the most common types of RGB-D cameras based on structure light mainly are the Microsoft Kinect-1, XtionProLive, Intel RealSense, and PrimeSense Carmine. Unlike the RGB-D cameras based on the principle of structure light (also shown in Table 1), Kinect-2 is the prominent representation of the RGB-D sensors based on ToF. Kinect-2 measures the depth by calculating the time delay between the continuously modulated harmonic signal and the received signal [10,11]. According to the time delay value and speed of light, the depth is equal to half the product of the time delay and speed of light. Usually, the depth measurements are sensitively and easily affected by multiple factors [12,13]. Many practical applications often need to fuse depth and color information exactly. Therefore, the accurate relative parameters between depth and RGB cameras are very critical to RGB-D cameras [14]. It is a premise and an important step to calibrate an RGB-D camera before implementing particular applications. When the resolutions of both depth and color images are not equal to each other, the relative pose between a depth camera and an RGB camera calibrated by the stereo calibration possibly includes a deviation of registration. Moreover, in most existing correction models of depth, most researchers calibrated the depth measurements by using the reference depths measured from extra measuring equipment or establishing the error models of depth. However, not all error models of depth could be applied to calibrate kinds of RGB-D sensors, and the reference depths obtained by extra measuring equipment inevitably still had a few errors. Consequently, in view of the above-mentioned facts, we established the rigorous mathematics relationship model between a depth camera and an RGB camera based on the collinear equation and space resection. Thus, the relative pose between depth and RGB cameras and the depths were calibrated based on a 3D control field in this paper.

RGB-D camera calibration has attracted extensive research and focus. Many calibration models have been published ever since. The earlier researchers considered that the corners of a checkerboard in depth and color images were coplanar to each other when depth and RGB images were captured by Kinect-1 simultaneously [15,16]. Therefore, the coplanar geometry property was used to calibrate the relative pose between a depth camera and an RGB camera. The depth values were all considered as a linear function of the actual depth [15,16]. However, the corners of a checkerboard in depth images needed to be marked manually, and the factor of distorted pixels in the depth image was not included in their consideration [15,16]. Due to the difficulty in identifying the corners of a checkerboard in a depth image directly, a semi-transparent checkerboard [17] and a board with the same circular holes [18,19] were selected as the calibration objects to help identify the corners for calibrating an RGB-D camera. Based on the geometric property that any three points in a 1D object are collinear, a 1D object [20] such as a stick was used to calibrate the RGB-D camera, too. In the existing calibration models of [17,18,19,20], the disadvantage was that the depth values could affect the accuracy of extracting corners in the calibration objects, and the depth noise was the main factor limiting the accuracy of the calibration. Moreover, the calibration objects utilized in the models of [18,19] needed some certain production requirements: the smaller sizes of objects could not be detected accurately when depth images were captured at far distance, and it might lead to a smaller number of corners being obtained at close range. Compared to the accuracy and practical operability of the calibration models presented in [15,16,17,18,19,20], the calibration model based on a spherical object [21] was proposed with a strong maneuverability and robustness to depth noise. However, the centers of spherical objects in an RGB image or depth image were obtained by applying the theory of Hough transform. From our experience, whether obtaining the accurate centers of a spherical object or not depended on the consistent texture information on its surface and the view of capturing the depth image. Generally, after obtaining the intrinsic parameters of both a depth camera and an RGB camera, the relative parameters between the two cameras were calibrated by a stereo calibration model. However, when the resolutions of both depth and color images were not equal to each other, which unfortunately happened often, the model of stereo calibration might not work well. Chen [22] was the first to calibrate the relative pose between an infrared camera and a color camera by introducing a weight based on the resolution ratio of an infrared image and a color image. The relative pose between an infrared camera and an RGB camera was solved by minimizing the reprojection errors of infrared images and color images. The calibration results of the relative parameters were not provided in his paper. The common limitation of the above-mentioned methods was that they only focused on the estimation of rigid transformation between a depth camera and an RGB camera, but the error of depth measurement was hardly considered.

For depth correction, Smisek [23] was the first to specify the distortion in depth correction, and observed that the Microsoft Kinect-1 exhibited residuals in a close range of depth measurements. Kim [24] introduced the theory of weighted joint bilateral filtering based on distance transform between color and depth images to calibrate depth. However, the models mentioned above hardly addressed depth correction at far distances. Moreover, the infrared and RGB cameras were regarded as binocular models to correct the depth measurements. Canessa [25] proposed a quadratic polynomial model to calibrate the depth measurement of pre-pixels in a depth image. This method needed a higher requirement for the placed position of a checkerboard when correcting depth at far distances, and the range of depth correction was from 0.6 m to 2.0 m roughly. When considering the infrared and RGB camera as a binocular model, incorrect features matching or the baseline between infrared and color cameras might also affect the accuracy and efficiency of depth correction.

Lindner [26,27] and Fuchs [28] applied the cubic B-spline function to fit the depth error, but the reference depths utilized in their models were either of lower accuracy [27] or were measured by the extra measuring equipment with a higher cost [26,28]. The polynomial model combined with the simple 3D control field [29] was also proposed to calibrate the depth error, while the accuracy of depth correction depended deeply on the identification of targets, and the robustness of the polynomial model was not verified. Jung [30] proposed calibrating the depth error by making the calibration board with 2.5 D patterns, but the correction model of depth deeply depended on the accuracy of fitting the centers of the patterns. In the case of a far range, the 2.5 D patterns in depth images could be too small to be used for identifying the centers exactly. Some researchers [31,32] considered the depth error as a random error, and they established the error model of depth correspondingly. Chen [33] adopted the heteroscedastic Gaussian distribution to establish the error model of a depth measurement and corrected the depth via an auxiliary system with higher cost. As a matter of fact, it was fairly difficult to develop a suitable error model of depth to describe the depth error. It was not reasonable to consider the depth error as a random error, either. In terms of the theory of machine learning with the ability to model in complex problems, it was also used to model and calibrate depth [34,35]. When depth correction was modeled based on machine learning, massive data would be used to obtain the training dataset. Sometimes, the effect of the training model might cause a phenomenon of overfitting. As RGB-D cameras were applied in the SLAM system widely, the multiplier of depth was proposed to correct the depth measurements via the SLAM system [36,37,38]. To the best of our knowledge, these types of methods require solving numerous parameters and larger amounts of computation.

Herrera et al. [39] established an exponential model based on disparity images to correct the depth error with a higher accuracy, and the relative displacement of both a depth camera and an RGB camera was also estimated. Ever since, many improvements were made to simplify this exponential model and improve the calibration accuracy. The improvements over the work of Herrera [39] were included: using the first order of the Taylor series instead of the exponential function to denote the model of distorted disparity [40], modifying the calibration pipeline of Herrera [39] to avoid drift of depth [41], or selecting the cuboids with geometric constraint conditions as calibration objects to calibrate an RGB-D camera [42]. To obtain a better calibration result, researchers calibrated the distorted disparity images and then acquired the actual depths by calculating the conversion coefficients between the depths and values of disparity [43,44]. However, such models could only be suitable for the RGB-D cameras based on structure light. Different from the methods to calibrate depths by using disparity images, other researchers believed that the error of depth measurement was caused by the factor of distortion from both an infrared emitter and an infrared camera [45]. Based on this point, the depth could be corrected by calibrating the distortion component [45] or the relative pose [46] between the infrared emitter and infrared camera, respectively. Combining this method with the research studies on the depth correction in [45,46], the researchers [47,48] calibrated the depth measurements by optimizing the distortion and relative pose between an infrared emitter and infrared camera simultaneously. However, the methods could only calibrate the system error existing in a depth camera. Meanwhile, it was not easy to converge in the optimizing process when solving larger numbers of parameters.

Considering the advantages and limitations of the models mentioned above comprehensively, it was difficult to calibrate the relative pose along with the depth based on the calibration artefact or checkerboard. Due to some correction models for depth that were proposed to calibrate the sensors based on structure light only, the models were not suitable for other kinds of RGB-D cameras based on ToF. In addition, most existing methods adopted the stereo calibration model to calibrate the relative pose between a depth camera and an RGB camera, whose outcomes could not be good in case of different sizes of RGB and depth images. According to [7], we proposed the strict imaging model of an RGB-D camera based on the collinear equation. Combining with our 3D control field, the relative parameters between depth and color cameras were solved by space resection using 3–5 infrared and RGB images. For depth correction, we could easily identify the targets of infrared images in short or far distance, and then we calculated the reference depths based on a 3D control field to calibrate the depth measurements. The details of our proposed method are presented in Section 2.

## 2. Methodology

**Figure 2:**
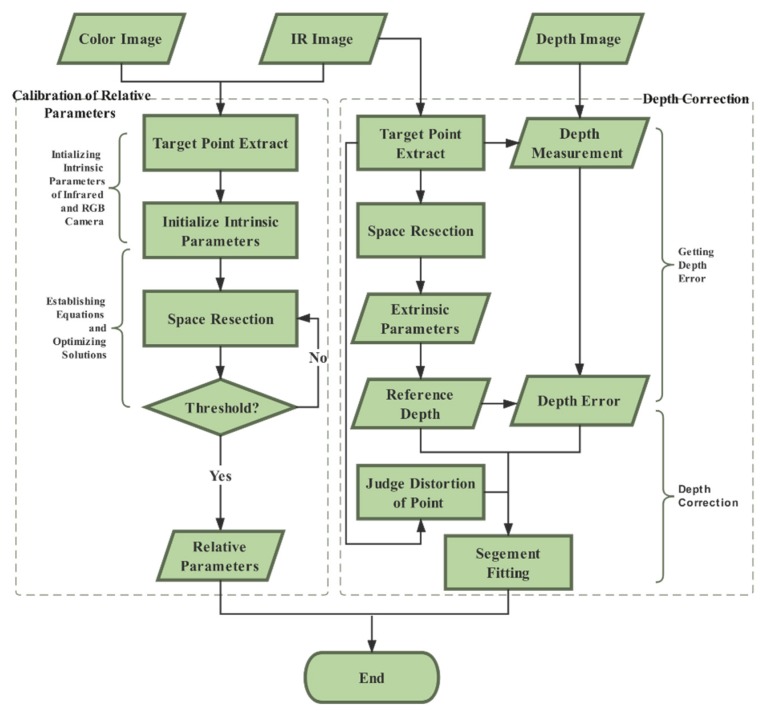
An overview of our proposed calibration method.

In this section, we explain our proposed calibration scheme in detail. We established a geometric imaging model of RGB-D camera based on a collinear equation, and then calibrated the depth measurement according to the rigorous imaging model of a depth camera. For the relative pose between a depth camera and an RGB camera, we built the calibration model based on space resection and collinear equation in the field of photogrammetry, and optimized the solution of relative pose by least-squares iteration. Both aspects constituted our calibration scheme in this paper. The flowchart of our proposed method is shown in Figure 2. We present our method and process systematically in Section 2.1 and Section 2.2, respectively.

### 2.1. Geometric Calibration Model of RGB-D Sensor

Camera calibration is a process of establishing the corresponding relationship model between points in both object space and image space [49], and solving the parameters of an imaging geometric model of a camera [50,51]. RGB-D camera calibration includes the calibration of an RGB camera and a depth camera. The intrinsic parameters of an RGB or depth camera include the distorted coefficients, as well as the intrinsic matrix *K* with the focal length (*f*_x_,*f*_y_) and principal point (*c*_x_,*c*_y_). According to the pinhole model [49], the relationship model between a pixel and the corresponding point in a camera coordinate system is modeled as shown in Equation (1). Meanwhile, the pixel and point in a world coordinate system are also formulated with the rotation matrix *R* and translation vector *t* listed in Equation (2). For camera distortion correction, the model of Brown [52] is often adopted to correct the distorted pixels as shown in Equation (3), where (x,y) represent the corrected points; *r*^2^ is equal to the sum of *x*^2^ and *y*^2^; and (*x_d_*,*y_d_*) indicates the corresponding distorted point.

(1) $$\left(\begin{array}{c} u\\ v\\ 1 \end{array}\right)=\frac{1}{Z}\left(\begin{array}{ccc} f_{x} & 0 & c_{x}\\ 0 & f_{y} & c_{y}\\ 0 & 0 & 1 \end{array}\right)\left(\begin{array}{c} X_{c}\\ Y_{c}\\ Z_{c} \end{array}\right)\;=\;\;\frac{1}{Z}\mathrm{KP}$$

(2) $$Z\left(\begin{array}{c} u\\ v\\ 1 \end{array}\right)=K\left(RP_{w}+t\right)=KTP_{w}$$

(3) $$\{\;\begin{array}{c} x_{d}=\;x\left({1+k}_{1}r^{2}{+k}_{2}r^{4}{+k}_{3}r^{6}\right)+{2p}_{1}{\mathrm{xy}+p}_{2}\left(r^{2}{+2x}^{2}\right)\\ y_{d}{=\;y(1+k}_{1}r^{2}{+k}_{2}r^{4}{+k}_{3}r^{6}{)+\mathrm{yp}}_{1}r^{2}{+2p}_{1}y^{2}{+2p}_{2}\mathrm{xy} \end{array}$$

**Figure 3:**
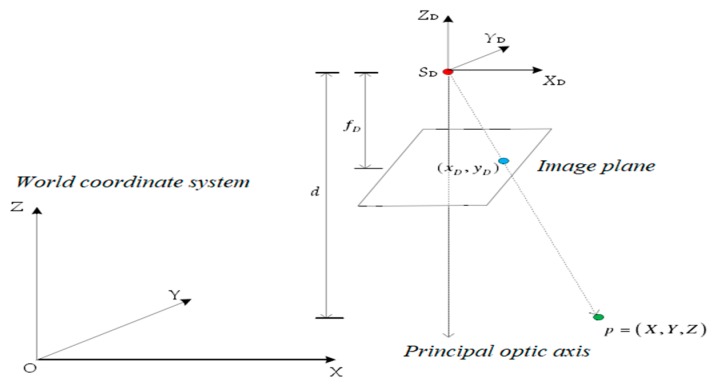
The imaging principle of a depth camera.

As both the RGB camera and depth camera followed the pinhole mode, we assumed that any one point in the world coordinate system O-XYZ was P(X,Y,Z). Its corresponding image point coordinate was ($x_{D}$, $y_{D}$) in a depth image. *f_D_* was the focal length of the depth camera. The origin of the depth camera coordinate system S_D_-X_D_Y_D_Z_D_ was defined in the optical center of the depth camera, where the *X*-axis was vertical to the *Z*-axis and pointing to the right. The direction of the *Z*-axis was defined in the opposite direction of the principal optical axis. Three axes were conformed to the right-hand criterion. The imaging principle of a depth camera and the description of each coordinate system are shown in Figure 3. Assuming that *R^D^* was the rotation matrix of a depth camera coordinate system relative to the world coordinate system O-XYZ, the origin of S_D_-X_D_Y_D_Z_D_ in the coordinate system O-XYZ was denoted as T^D^(X_S_,Y_S_,Z_S_). According to a collinear equation, the relationship between any points of object space and the corresponding image point is expressed as in Equation (4):

(4) $$\left(\begin{array}{c} X-Xs\\ Y-Ys\\ Z-Zs \end{array}\right)=\lambda R^{D}\left(\begin{array}{c} x_{D}\\ y_{D}\\ -f_{D} \end{array}\right)$$

where *λ* was the scale factor, *d* was equal to *λ*, *f_D_* and *f_D_* was the focal length. Therefore, Equation (4) could also be rewritten as:

(5) $$\left(\begin{array}{c} X-Xs\\ Y-Ys\\ Z-Zs \end{array}\right)=\left(\begin{array}{ccc} R_{11} & R_{12} & R_{13}\\ R_{21} & R_{22} & R_{23}\\ R_{31} & R_{32} & R_{33} \end{array}\right)\left(\begin{array}{c} \lambda x_{D}\\ \lambda y_{D}\\ -d \end{array}\right)$$

By expanding Equation (5), (*x_D_*,*y_D_*) and *d* could be expressed respectively as follows:

(6) $$\left\{ \begin{array}{c} x_{D}=-f_{D}\frac{R_{11}\left(X-Xs\right)+R_{21}\left(Y-Ys\right)+R_{31}\left(Z-Zs\right)}{R_{13}\left(X-Xs\right)+R_{23}\left(Y-Ys\right)+R_{33}\left(Z-Zs\right)}\\ y_{D}=-f_{D}\frac{R_{12}\left(X-Xs\right)+R_{22}\left(Y-Ys\right)+R_{32}\left(Z-Zs\right)}{R_{13}\left(X-Xs\right)+R_{23}\left(Y-Ys\right)+R_{33}\left(Z-Zs\right)}\\ d=-[R_{13}\left(X-Xs\right)+R_{23}\left(Y-Ys\right)+R_{33}\left(Z-Zs\right)] \end{array}\right.$$

Thus, we could obtain the truth depth by Equation (6), which was used to correct the depth error.

In order to fuse the color and depth information, we needed to calibrate the relative parameters between the depth camera and RGB camera precisely. According to the space resection of a single image, we established the mapping relationship between the RGB and depth cameras. Firstly, the point P of object space in the depth image coordinate system was (x_D_,y_D_), and the 3D coordinate of P in the depth camera coordinate system was denoted in Equation (7). We also assumed that the relative rotation matrix of both the RGB and depth cameras was R and T( $X_{s}^{\prime }\;$, $\;Y_{s}^{\prime }\;$, $\;Z_{s}^{\prime })$ was the origin coordinate of the RGB camera in the depth camera coordinate system. We assumed that the coordinate of a point in the image space coordinate system was (*x_rgb_*,*y_rgb_*,−*f*_rgb_), and *f*_rgb_ was the focal length of the RGB camera. According to the collinear equation, the relationship model between the RGB and depth cameras was expressed in Equation (8). Considering the distortion of pixels in the RGB camera, Equation (8) was rewritten as Equation (9) too, where the principal point was denoted by (*x*_0_,*y*_0_); the corresponding pixel in the color image was (*x*_r_,*y*_r_), and (∆*x*,∆*y*) indicated the distortion components of the image point.

(7) $$\{\begin{array}{c} X_{D}=\frac{x_{D}d}{f_{D}}\\ Y_{D}=\frac{y_{D}d}{f_{D}}\\ Z_{D}=-d \end{array}$$

(8) $$\left\{ \begin{array}{c} x_{rgb}=-f_{rgb}\frac{R_{11}\left(X-X_{s}^{\prime }\right)+R_{21}\left(Y-Y_{s}^{\prime }\right)+R_{31}\left(Z-Z_{s}^{\prime }\right)}{R_{13}\left(X-X_{s}^{\prime }\right)+R_{23}\left(Y-Y_{s}^{\prime }\right)+R_{33}\left(Z-Z_{s}^{\prime }\right)}\\ y_{rgb\;}=-f_{rgb}\frac{R_{12}\left(X-X_{s}^{\prime }\right)+R_{22}\left(Y-Y_{s}^{\prime }\right)+R_{32}\left(Z-Z_{s}^{\prime }\right)}{R_{13}\left(X-X_{s}^{\prime }\right)+R_{23}\left(Y-Y_{s}^{\prime }\right)+R_{33}\left(Z-Z_{s}^{\prime }\right)} \end{array}\right.$$

(9) $$\left\{ \begin{array}{c} x_{r}-x_{0}-\Delta_{x}\;=-f_{x}\frac{R_{11}\left(X-X_{s}^{\prime }\right)+R_{21}\left(Y-Y_{s}^{\prime }\right)+R_{31}\left(Z-Z_{s}^{\prime }\right)}{R_{13}\left(X-X_{s}^{\prime }\right)+R_{23}\left(Y-Y_{s}^{\prime }\right)+R_{33}\left(Z-Z_{s}^{\prime }\right)}\\ y_{r}-y_{0}-\Delta_{y}\;=-f_{y}\frac{R_{12}\left(X-X_{s}^{\prime }\right)+R_{22}\left(Y-Y_{s}^{\prime }\right)+R_{32}\left(Z-Z_{s}^{\prime }\right)}{R_{13}\left(X-X_{s}^{\prime }\right)+R_{23}\left(Y-Y_{s}^{\prime }\right)+R_{33}\left(Z-Z_{s}^{\prime }\right)} \end{array}\right.$$

(10) $$(\begin{array}{c} X_{1},Y_{1},\;Z_{1},1,\;0,\;0,\;0,\;0,\;x_{1}X_{1},x_{1}Y_{1},x_{1}Z_{1}\\ 0,\;0,\;0,\;0,\;X_{1}\;,Y_{1},Z_{1},\;1,\;y_{1}X_{1},y_{1}Y_{1},y_{1}Z_{1}\\ \vdots ,\;\vdots ,\;\vdots ,\;\vdots ,\;\vdots ,\;\vdots ,\;\vdots ,\;\vdots ,\;\vdots ,\;\vdots ,\;\vdots \\ X_{6},Y_{6},\;Z_{6},1,\;0\;,0,\;0,\;0,\;x_{6}X_{6},x_{6}Y_{6},x_{6}Z_{6}\\ 0,\;0,\;0,\;0,X_{6},Y_{6}\;,Z_{6},\;1,\;x_{6}X_{6},x_{6}Y_{6},x_{6}Z_{6} \end{array})(\begin{array}{c} l_{1}\\ l_{2}\\ \begin{array}{c} \vdots \\ \vdots \\ l_{11} \end{array} \end{array})+(\begin{array}{c} x_{1}\\ y_{1}\\ \begin{array}{c} \vdots \\ x_{6}\\ y_{6} \end{array} \end{array})=0$$

**Figure 4:**
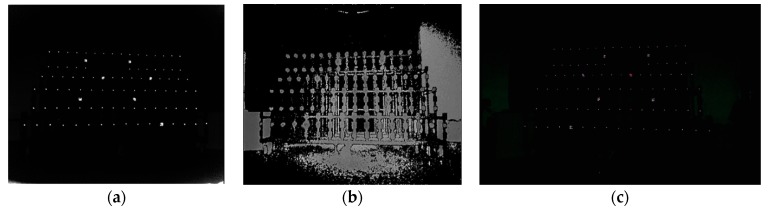
Images captured in a 3D control field: (**a**) infrared image; (**b**) depth image; (**c**) RGB image.

In this paper, when calibrating the intrinsic parameters of the depth camera or RGB camera by a 3D control field (shown in Figure 8a), we applied direct linear transformations [53] denoted by Equation (10) to calculate the intrinsic parameters. In Equation (10), $l_{i}\left(i=1,2,3,\cdots 11\right)$ was the function related to the intrinsic parameters and extrinsic parameters, respectively. Both the principal point (*x*_0_,*y*_0_) and focal length (*f_x_*,*f_y_*) were included in the 11 coefficients, and we solved the intrinsic parameters of the depth or RGB camera in six targets at least. We also could calibrate the intrinsic parameters of both the depth and RGB cameras in the method of [54]. As we had accurate 3D coordinates of target points in the control field and the corresponding pixels in infrared images, we could calculate the extrinsic parameters between the depth camera and 3D control field based on space resection. Thus, the 3D coordinates of each target point in the depth camera coordinate system were calculated, too. Meanwhile, we also extracted the pixels of the target points in the color images. Since the target points could not be detected in the depth images (shown in Figure 4b), we selected the infrared images instead (shown in Figure 4a). Finally, considering the distorted components of the pixels in the color image, we established equations based on Equation (9) using the pixels of color images and their corresponding 3D coordinates in a depth camera coordinate system. The relative parameters between the depth camera and RGB camera were solved by using the method of iterative least squares.

### 2.2. Depth Correction by 3D Control Field or Checkerboard

Depth calibration is always the most important research content of an RGB-D camera. Below are the detailed processes to calibrate the depth measurements. As we could obtain the accurate 3D coordinates of target points in a 3D control field, the corresponding pixels in an infrared image, and the focus length of the depth camera, we calculated the extrinsic parameters of each infrared image relative to the 3D control field by the theory of space resection of a single image formulated in Equation (9). We could also calculate the extrinsic parameters relative to a checkerboard. Based on the coordinates of the corners of a checkerboard and the corresponding pixels of an infrared image, we established equations based on Equation (9) to optimize the extrinsic parameters between the infrared camera and the checkerboard. Based on Equation (11), the reference depth of each depth measurement in the depth image was calculated by using the extrinsic parameters. Considering that the depth errors varied inconsistently with different depth measurements [55], we applied the most appropriate function model to fit the depth errors. To improve the accuracy of the depth correction, the distortion of pixels with different degrees in the depth image were considered. We divided the distortion of pixels in the depth image into two parts (see Section 3.2 for details): obvious distortion and slight distortion. As the size of the pattern in the checkerboard was smaller, we directly calibrated the depth by the method of function fitting. To correct the depth measurements better, we applied a 3D control field to calibrate the depths by combining it with the distortion of pixels in the depth camera in this paper.

When calibrating the depth measurements via a 3D control field or checkerboard, the RGB-D camera was headed toward the 3D control field or checkerboard. The sensor was placed at different distances ranging from 500 mm to 4500 mm with a fixed size of steps. When we acquired infrared and depth images simultaneously, the infrared image set $\left\{IR_{n}\right\}$ and depth image set $\left\{Dep_{n}\right\}$ were formalized. Both the depth and infrared images were taken within the available ranges of depth. The target points of the 3D control field or corners in the checkerboard were detected on infrared images, and their 3D coordinates had been provided in advance. The space resection was used to calculate the extrinsic parameters between the depth images and the 3D control field or checkerboard. Finally, the corresponding depths of the target points in the depth camera coordinate system were calculated by Equation (11) and served as the reference depths. We compared them with the depth measurements and fitted the errors of different depths by using the most appropriate functional model.

(11) $$\left(\begin{array}{c} X\\ Y\\ Z \end{array}\right)=R\left(\begin{array}{c} X_{D}\\ Y_{D}\\ Z_{D} \end{array}\right)+T$$

## 3. Experiment and Results

### 3.1. Calibration of Relative Extrinsic Parameters of RGB-D Camera

In this paper, taking Kinect-2 as an example, the resolution of an RGB image was 1920 × 1080, and that of both the depth image and infrared image were 512 × 424. There were about 90 targets including several larger reflective targets distributed in the 3D control field evenly (Figure 4a) which were made of welded steel structure. The smaller reflective markers were tagged in the fixed places. The larger reflective markers (seen in Section 3.2) were also set in a 3D control field, which were used to identify and register the small target points in the RGB or infrared image conveniently. We applied a 3D control field to initialize the intrinsic parameters of Kinect-2 in the method of [54]. In theory, we obtained a group of intrinsic parameters with a higher accuracy by adopting only one RGB or infrared image captured in the 3D control field. In order to obtain more accurate results, three to five RGB and infrared images with high quality were selected to calibrate the two cameras. After getting the intrinsic parameters of the depth and RGB cameras, we applied the relative parameters of the calibration model introduced in Section 2.1 to calculate the extrinsic parameters of the depth camera system related to the 3D control field. The 3D coordinates of the target points in the depth camera system were calculated by Equation (11). Considering the distorted components denoted in Equation (9), we established more than 540 equations by using the pixel coordinates of the target points in RGB images and the corresponding 3D coordinates of target points in the depth camera coordinate system. The initial values of the relative parameters were set to 0, and then we acquired the optimal solution after the third iterative least squares.

According to the comparison of different calibration methods for RGB-D sensors discussed in [9], the best calibration parameters of Kinect-2 were obtained in the method of Bouguet [56], which was verified and concluded in the paper of [9]. To compare with the accuracy of the relative parameters calibrated by our calibration model, we also calibrated Kinect-2 by using the method of Bouguet [56]. We registered the depth and color images and compared the registration results. The experimental results are presented in Section 3.3. The coordinates of target points in the 3D control field were fairly accurate, and the reprojection errors of both Bouguet [56] and our method were less than 0.4 pixels. The intrinsic parameters and relative parameters of Kinect-2 calculated by our proposed model and Bouguet [56] are shown in Table 2 and Table 3, respectively. We selected several groups of infrared and RGB images taken in the 3D control field. We extracted the corresponding pixels of the target points in the infrared and RGB images precisely. Using the calibrated relative parameters between the depth camera and RGB camera, we calculated the reprojection errors by projecting pixels from infrared images to RGB images rigidly. The mean reprojection errors of our calibration model and Bouguet [56] are presented in Table 3, too.

### 3.2. Depth Correction

Currently, although models of depth correction have been proposed, there are still key limitations in these models. For example, because the distortion of pixels was related to the depth error, it was not appropriate to fit the depth error without considering different degrees of distortion, such as [26,29]. Moreover, in the models proposed in [28,33], the reference depth was measured by extra measuring equipment, but the measuring equipment itself had errors inevitably. Finally, some correction models of depth were proposed only to calibrate RGB-D cameras based on structure light [39,41,42,44], which were not suitable for the RGB-D sensors based on ToF and the Microsoft Kinect-2, for instance. In this paper, our proposed model was based on the space resection and collinear equation to calibrate depth with considering of the distortion of pixels, and our model could overcome these above-mentioned disadvantages. With the rigorous mathematical formulas in our calibration model, the reference depth calculated by Equation (6) was accurate without the aid of measuring equipment.

**Figure 5:**
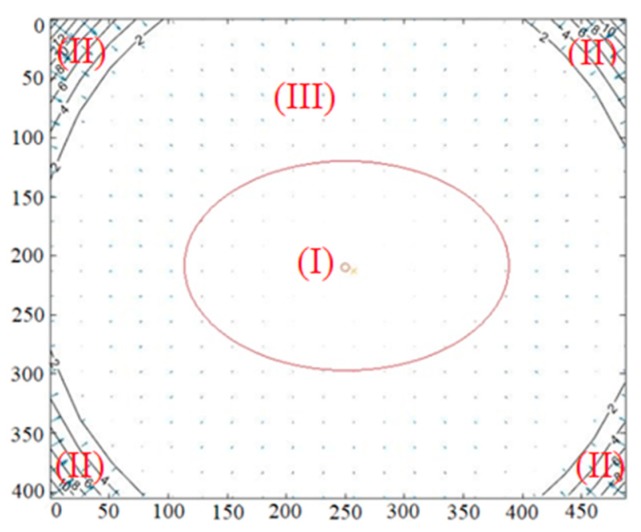
Complete distortion effect of the infrared camera.

According to the overall distortion effect of the infrared camera shown in Figure 5, we divided the distortion of pixels in the image into two parts: obvious distortion and slight distortion. It was more suitable to correct the depth error based on the different distorted regions in the depth image. The pixels in the red ellipse had a slight distortion, whereas others had an obvious distortion. According to the previous assessment of depths and analysis of distorted pixels in the depth camera [31,55], we knew that the farther away a pixel was from the principal point of an image, the more obvious its distortion was. Combining with the overall distorted fact of pixels in an infrared camera, we briefly divided the ranges of pixels into three regions (shown in Figure 5): the region of the red ellipse (I), the region of four corners (II), and a region consisting of the rest, except for the central region and the four corners (III). As there was slight distortion in region (I), we assumed that the pixels in region (I) were affected by the distortion negligibly in the depth measurement. In addition, the number of pixels with valid depth measurements in region (II) was smaller, and the distortion of pixels in both regions (II) and (III) were more obvious. Thus, we merged region (II) and region (III), and we considered that the pixels with depth measurements in the two regions were affected by the distortion significantly. The interval ranges of the red ellipse were approximately *x* ∈ (165, 334) and *y* ∈ (141,279), respectively.

**Figure 6:**
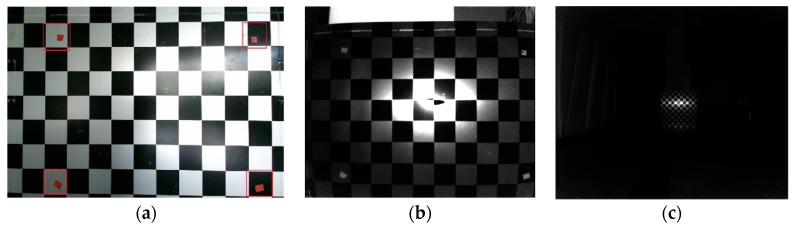
Image of checkerboard and infrared image: (**a**) checkerboard; (**b**) infrared image captured at a distance of 0.5 m; (**c**) infrared image captured at a distance of 4.5 m.

We used both a checkerboard and a 3D control field to correct depth, respectively. The size of the checkerboard was 80 mm. We calibrated depth errors by the checkerboard at different depths. We pasted four simple markers in the checkerboard (shown in Figure 6a), forming a 9 × 7 grid area with 40 corner points in the region. We placed Kinect-2 in front of the checkerboard at the distance of 0.5 m firstly. Then, we started moving Kinect-2 with the fixed step of 0.1 m and captured both infrared and depth images simultaneously. After the extrinsic parameters of infrared images related to the checkerboard were calculated by theory of space resection, the reference depth was obtained by Equation (11). The infrared images of the checkerboard captured at a short (0.5 m) and far distance (4.5 m) are shown in Figure 6b,c.

**Figure 7:**
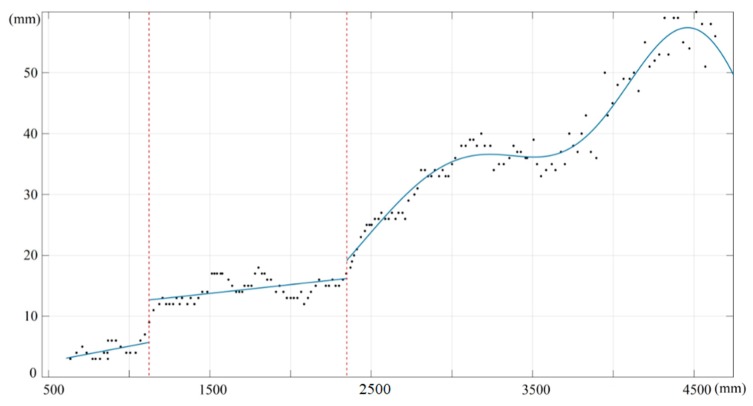
Fitting curve of the depth error at different ranges of depth.

After getting the reference depths of corners in infrared images, we could acquire the depth measurements from the corresponding depth images. The correction results of the depth measurements are shown in Figure 7. We considered the altitude of depth error as the vertical axis and the depth value as the horizontal axis to solve the fitting function. To ensure the effect of error fitting, the depths were divided into three intervals: 0.5–1.2 m, 1.2–2.3 m, and depths that were greater than 2.3 m. In order to ensure that the root mean square standard error of the fitting result was minimal in each interval, both linear and Gaussian function models were used to fit the depth error, respectively. Reviewing the fitting results, all of the root mean square standard errors (RMSE) of the fitting function were less than 2.25 mm.

**Figure 8:**
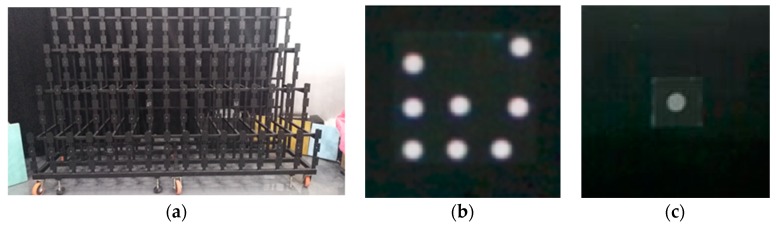
3D control field and reflective target: 3D control field (**a**); large reflective target (**b**); small reflective target (**c**)**.**

**Figure 9:**
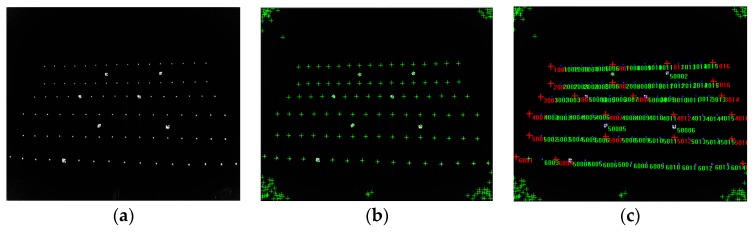
Image captured in 3D control field (**a**); (**b**) reflective targets of infrared image were identified; (**c**) reflective targets of infrared image were registered.

We also corrected the depth by means of a 3D control field (Figure 8a). The artificial backlight target had strong reflectivity (Figure 8b,c), and it was convenient and accurate to extract the pixels of the target points in the infrared image. According to the pixels of the target points in an infrared image and the 3D coordinates in the control field, the extrinsic parameters of each infrared image related to the 3D control field were calculated by our method described in Section 2. Then, the rotation matrix and translation vector were calculated, and the reference depths were obtained by Equation (11). Finally, having obtained the reference depths and depth measurements, we calculated and fitted the depth errors by applying a suitable function model. The specific calibration process and steps were as follows:

(1) We illuminated the 3D control field using an extra light source, and then we captured infrared and depth images at different depths (Figure 9a). When taking infrared images, we tried to ensure that the reflective targets were evenly distributed in the images. For detecting the small reflective targets readily, we also ensured that there were several large reflective targets existing in the infrared images.
(2) According to our procedure of identifying the reflective targets, the pixels of reflective targets in infrared images were automatically identified and extracted (as shown in Figure 9b). The coordinates of the 3D control points were also registered (as shown in Figure 9c).
(3) The reference depths from the target points to the center of the infrared camera were calculated by Equations (6) and (11). According to the pixel coordinates in each infrared image, the depth measurements were acquired in the corresponding depth image. We calculated the depth errors and calibrated the depths by applying the most suitable function model.

**Figure 10:**
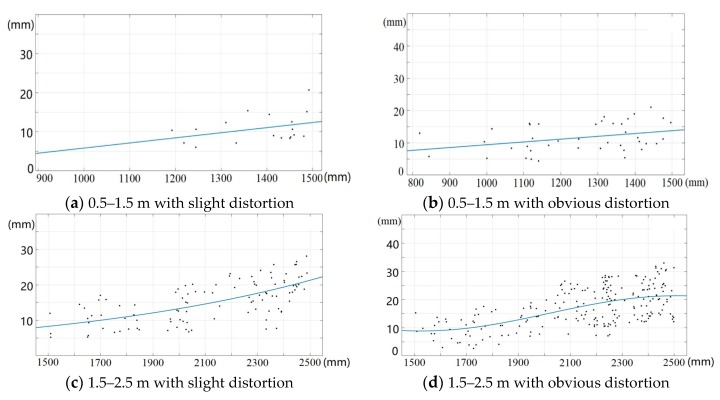

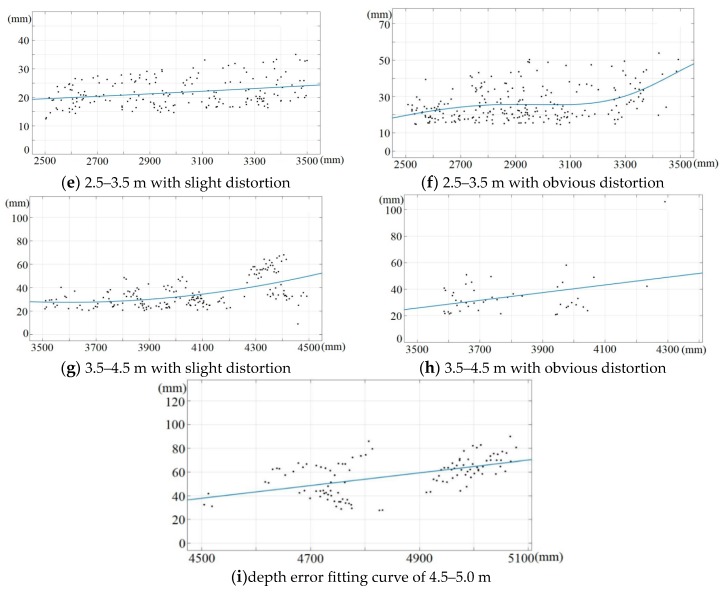
The results of fitting depth error combining with pixel distortion.

In the process of depth correction, according to the distorted fact of pixels in the infrared camera (shown in Figure 5), we calculated the distance from each pixel point to the principal point of image and determined the pixel into slightly distorted or obviously distorted areas by judging the value of ***r*** in Equation (12). We corrected the depth errors by the method of segmental fitting according to different degrees of distortion of pixels. The 3D control field was observed by Kinect-2 at different distances from 0.5 m to 5.0 m with a regular step equal to 0.2 m. For the infrared images of targets obtained by Kinect-2, R and T were calculated by space resection. The reference depths of the target points in the depth camera coordinate system were calculated by Equation (11). According to the ranges of valid depth, we divided four fitting intervals with every fixed distance of 1.0 m. According to the value of ***r*** in Equation (12), each fitting interval was also divided into the regions with obvious distortion and slight distortion. When the depth measurements were greater than 4.5 m, the depth errors were significant. Thus, we directly adopted a function model to fit and correct the depth measurements. The depth correction results are shown in Figure 10.

(12) $$r\;=\;(\frac{x}{f_{x}}{-c}_{x}{)}^{2}+(-\frac{y}{f_{y}}{+c}_{y}{)}^{2}$$

### 3.3. Validation of Calibration Model

To examine the performance of depth correction and the relative pose between the depth camera and RGB camera, we collected several groups of depth images and color images in our laboratory. We registered depth and color images by applying our calibration parameters and the calibration parameters calibrated by Bouguet [56]. Then, we compared our results with those obtained by using the relative parameters from Bouguet [56]. For the validation of depth correction, we compared the performance of 3D reconstruction before depth correction with that after depth correction.

**Figure 11:**
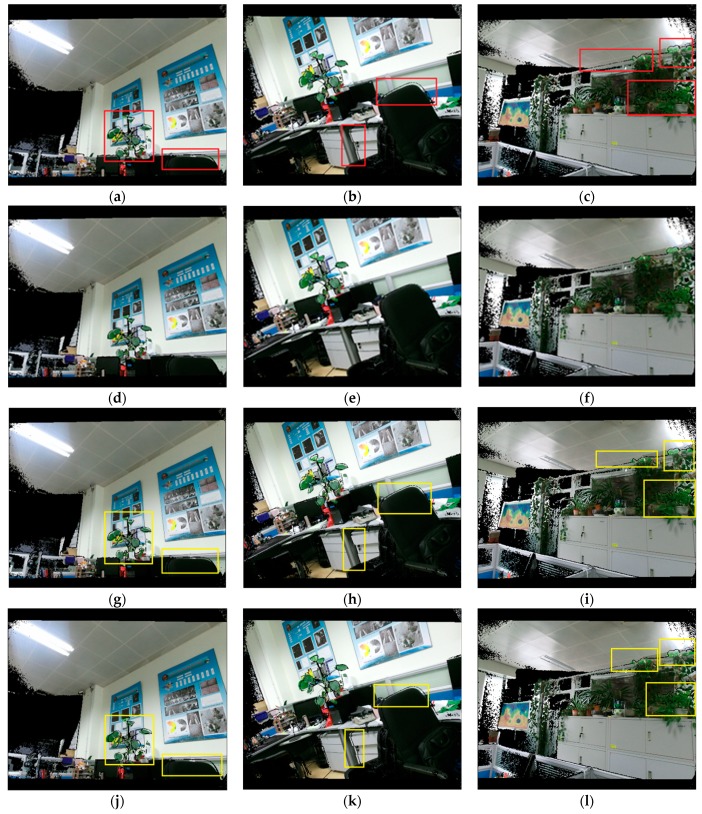
Registration results in different scenes: registration results of different scenes obtained by our method (**d**–**f**); the corresponding results of [56] (**a**–**c**); registration results obtained by using the calibrated rotation angles in our model and the translation components of [56] (**g**–**i**); registration results by using the rotation angles of [56] and the translation components in our model (**j**–**l**).

We selected three groups of depth and RGB images captured in different scenes to verify the accuracy of relative parameters. The size of the depth image was 512 × 424, and size of the color image was 1920 × 1080. The registration results obtained by our calibration parameters are shown in Figure 11d–f, and the comparison results obtained by [56] are presented in Figure 11a–c. The registration objects marked in red boxes showed the visible deviations in Figure 11a–c, compared to the corresponding objects in Figure 11d–f. Comparing to the registration results shown in Figure 11a–c, we found that the results obtained by our calibration model had a dramatic improvement in the performance in representing the edge of the objects.

After analyzing the experimental results and calibration parameters carefully, we concluded that angular elements in the relative parameters were the main factor that affected the accuracy of the registration. We tried to replace the angle elements obtained by Bouguet [56] with those from our model. The registration results are shown in Figure 11g–i. We found that the registration results had obvious improvements compared to the registration results shown in Figure 11a–c. However, when replacing the translation parameters with those from our model, the present results in Figure 11j–l did not improve obviously. Therefore, we believed that the angular elements of the relative parameters were the main factor affecting the accuracy of the relative parameters between the depth camera and RGB camera. One of the main advantages in our model compared to Bouguet [56] was that our proposed method had few relevant parameters to solve but returned better outcomes with higher accuracy. As a matter of fact, our model needed to solve and optimize only six relevant parameters, while the model of Bouguet [56] needed to optimize 22 parameters simultaneously. A large numbers of parameters might lead to a lower accuracy of solutions by nonlinear optimization. As a result, the calibration of the RGB-D camera performed with a higher accuracy in our method than in the method of [56].

**Figure 12:**
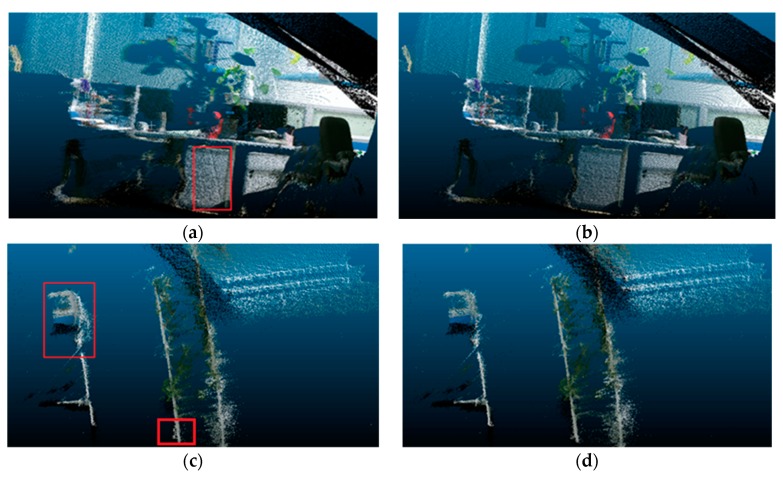
3D reconstruction before and after depth correction: Reconstruction before depth correction (**a,c**); Reconstruction after depth correction (**b,d**).

The performances of 3D reconstruction by using Kinect-2 before and after depth correction are shown in Figure 12. Figures on the left represented the performances of 3D reconstruction in two different scenes before depth correction, while those on the right displayed the performances of 3D reconstruction after depth correction. It could be clearly seen that the warp in the cupboard and wall marked by red squares were removed after depth correction in Figure 12b. Another example is presented in Figure 12c,d, showing a significant improvement of 3D reconstruction after depth correction, too. We also found that the distorted regions marked in red boxes in Figure 12c were eliminated in Figure 12d.

In order to quantitatively verify the correction model of depth, we selected the scene shown in Figure 12a for quantitative analysis. We applied Kinect-2 to reconstruct the scene in different depths and calculated the depth error before and after depth correction. We accurately measured the sizes of actual objects with regular shape in our lab, such as the width or height of the cabinets. Then, we measured the sizes of the same objects in the 3D point cloud before and after depth correction at different distances. The sizes of objects in the scene were measured several times before and after depth correction, and the averages were calculated. Then, the average errors were calculated by comparing with the actual sizes of objects and are shown in Table 4. It was easy to find that the performances of 3D reconstruction greatly improved after depth correction when the depth values were greater than 2.0 m. The errors were reduced by about 42% on average. However, the effect of depth correction was hardly obvious when the depths were less than 1.5 m. Based on previous research studies on the depth measurements of Kinect-2 [55], we concluded that the reason was that Kinect-2 was based on the principle of ToF to measure depth, and it had minor depth errors at close range and obvious errors at far distances.

## 4. Conclusions

In this paper, based on the theory of collinear equation and space resection, we established a new model to calibrate the depth and relative parameters between a depth camera and an RGB camera. Although the collinear equation and space resection have been familiar in the field of photogrammetry, we applied the theory in the RGB-D camera calibration and established the calibration model with rigorous mathematical formulas. Our calibration model included two parts: (1) the calibration of the relative parameters between the depth camera and the RGB camera, and (2) depth correction. The relative parameters obtained by our model enabled RGB-D cameras to perform better by overcoming the limitation of different resolutions of depth images and RGB images, which was the reason why we selected Kinect-2 as our research example to verify our proposed method. For depth correction, because Kinect-2 itself could achieve better accuracy in short distances without any depth correction [55], our correction model of depth might not achieve any significant improvements in the short distance. However, when it came to the far distance, greater than 2.0 m for instance, our method reduced the depth errors significantly and achieved an accuracy improvement of around 42% on average. Compared to most of the existing models for depth correction, our proposed model had another two advantages. (1) Our method could be applied to not only Kinect-2 based on the principle of ToF but also work on the sensors based on that of structure light. (2) Our correction model of depth was established with rigorous mathematical formulas; thus, the reference depth calculated by our model had few errors. Compared to the reference depth obtained by some correction models of depth, the reference depth was obtained by our model without the aid of extra measuring equipment. Future research studies should focus on the following two parts: (1) the regions of distortion of pixels in a depth camera should be divided more subtly; and (2) both a 3D control field and checkerboard should be adopted together to calibrate the depth error for acquiring a higher accuracy of depth correction at close and far ranges.

## Figures and Tables

**Table 1:** Configuration parameters of RGB-D cameras.

Attribute	Kinect-1	Kinect-2	XtionProlive	Real Sense	Carmine 1.08	Carmine 1.09
Field of angle (H × V)	57.5° × 43.5°	70° × 60°	58° × 45°	59° × 46°	57.5° × 45°	57.5° × 45°
Resolution of color (pix)	640 × 480	1920 × 1080	1280 × 1024	1920 × 1080	640 × 480	640 × 480
Frame rate of color image (fps)	30	30	30	30/60	30	30
Resolution of depth (pix)	320 × 240	512 × 424	640 × 480	640 × 480	640 × 480	640 × 480
Depth range (m)	0.8–4.0	0.5–4.2	0.8–3.5	0.2–1.2	0.8–3.5	0.35–1.4
Frame rate of depth image (fps)	30	30	30	30/60	60	60
Principle of depth	SL	TOF	SL	SL	SL	SL

**Table 2:** Intrinsic parameters of Kinect-2.

	Our Method		Bouguet [56]	
	Depth camera	RGB camera	Depth camera	RGB camera
[*f_x_*,*f_y_*] *pixel*	[365.60, 365.36]	[1055.47, 1055.15]	[364.84, 365.04]	[1056.37, 1055.96]
[*c_x_*,*c_y_*] *pixel*	[248.82, 208.63]	[940.58, 524.74]	[248.92, 209.84]	[940.60, 524.10]
[*k*_1_,*k*_2_, *p*_1_,*p*_2_]	[0.07923, –0.18888, –0.00016, –0.00002]	[0.04426, 0.03956, –0.00006, –0.00064]	[0.08188, –0.19272, –0.00033, 0.00007]	[0.04414, –0.03955, –0.00006, –0.00067]
[*Reprojection error*] *pixel*	0.176	0.225	0.221	0.319

**Table 3:** Relative extrinsic parameters of Kinect-2.

	Our Method	Bouguet [56]
[*Rotation angles*] *rad*	[0.00852, 0.00281, 0.0003550]	[–0.01209, –0.00107, 0.00379]
[*Translation*] *mm*	[51.45465, –0.72583, –3.21636]	[51.11498, –3.39875, –8.64573]
[*Reprojection error*] *pixel*	0.653	1.357

**Table 4:** Error of 3D reconstruction before and after depth correction.

Depth (mm)	Before Correction (mm)	After Correction (mm)	Percentage Reduction of Error
<1500	1.323	1.146	15.44%
(1500, 2500)	7.147	3.59	49.69%
(2500, 3500)	11.15	6.232	55.89%
(3500, 4500)	11.9	7.02	41.00%
>4500	18.84	9.20	51.17%
